# A Rapid Screening Assay for Clarithromycin-Resistant Mycobacterium avium Complex Using Melting Curve Analysis with Nonfluorescent Labeled Probes

**DOI:** 10.1128/spectrum.04326-22

**Published:** 2023-01-09

**Authors:** Akira Aoki, Hideto Jinno, Kenji Ogawa, Taku Nakagawa, Takayuki Inagaki, Takeaki Wajima, Yoshinori Okamoto, Kei-ichi Uchiya

**Affiliations:** a Department of Hygienic Chemistry, Faculty of Pharmacy, Meijo University, Nagoya, Japan; b Department of Respiratory Medicine, National Hospital Organization, Higashinagoya National Hospital, Nagoya, Japan; c Division of Pharmaceutical Sciences I, Faculty of Pharmacy, Meijo University, Nagoya, Japan; d Department of Microbiology, Faculty of Pharmacy, Meijo University, Nagoya, Japan; Johns Hopkins University School of Medicine

**Keywords:** *Mycobacterium avium* complex, clarithromycin resistance, melting curve analysis, nonfluorescent labeled probe, 23S ribosomal RNA, rapid test

## Abstract

Mycobacterium avium complex (MAC) thrives in various environments and mainly causes lung disease in humans. Because macrolide antibiotics such as clarithromycin or azithromycin are key drugs for MAC lung disease, the emergence of macrolide-resistant strains prevents the treatment of MAC. More than 95% of macrolide-resistant MAC strains are reported to have a point mutation in 23S rRNA domain V. This study successfully developed a melting curve assay using nonfluorescent labeled probes to detect the MAC mutation at positions 2058 to 2059 of the 23S rRNA gene (AA genotype, clarithromycin susceptible; TA, GA, AG, CA, AC, and AT genotypes, clarithromycin resistant). In the AA-specific probe assay, the melting peak of the DNA fragment of the AA genotype was higher than that of DNA fragments of other genotypes. Melting temperature (*T_m_*) values of the AA genotype and the other genotypes were about 80°C and 77°C, respectively. DNA fragments of each genotype were identified correctly in six other genotype-specific probes (TA, GA, AG, CA, AC, and AT) assays. Using genomic DNA from six genotype strains of M. avium and four genotype strains of M. intracellulare, we confirmed that all genomic DNAs could be correctly identified as individual genotypes according to the highest *T_m_* values among the same probe assays. These results indicate that this melting curve-based assay is able to determine MAC genotypes at positions 2058 to 2059 of the 23S rRNA gene. This simple method could contribute to the rapid detection of clarithromycin-resistant MAC strains and help to provide accurate drug therapy for MAC lung disease.

**IMPORTANCE** Since macrolide antibiotics such as clarithromycin or azithromycin are key drugs in multidrug therapy for Mycobacterium avium complex (MAC) lung diseases, the rapid detection of macrolide-resistant MAC strains has important implications for the treatment of MAC. Previous studies have reported a correlation between drug susceptibility testing and the mutation of macrolide resistance genes. In this study, we developed a novel melting curve-based assay using nonfluorescent labeled probes to identify both clarithromycin-resistant M. avium and M. intracellulare with mutations in the 23S rRNA gene, which is the clarithromycin or azithromycin resistance gene. This assay contributed to not only the detection of MAC mutations but also the determination of all genotypes at positions 2058 to 2059 of the 23S rRNA gene. Furthermore, because nonfluorescent labeled probes are used, this assay is more easily and more immediately available than other methods.

## INTRODUCTION

Nontuberculous mycobacteria (NTM) are ubiquitous in various environments, such as water, soil, and dust (1–3). NTM infection leads to various diseases, including lung diseases in humans and animals, and the number of patients with NTM lung disease is increasing worldwide (4, 5). Mycobacterium avium and Mycobacterium intracellulare are collectively referred to as M. avium complex (MAC), which is the most common lung disease among NTM. In the United States and Japan, MAC patients account for 60% or more of all NTM patients (6–9).

The macrolide antibiotics clarithromycin and azithromycin are key drugs for the treatment of MAC lung disease (10, 11). These drugs bind to the bacterial 23S rRNA in the 50S ribosomal subunit (12, 13). Macrolide-based multidrug therapy has been widely adopted to prevent the emergence of drug-resistant MAC. Clarithromycin and azithromycin are agents with dose-dependent effects on MAC lung disease. However, macrolide-resistant MAC sometimes emerges, which reduces treatment efficiency and increases mortality (14, 15). Therefore, the macrolide susceptibility test contributes to optimized drug therapy for MAC lung disease.

The culture-based method has been used as the clarithromycin susceptibility test (16). This conventional drug susceptibility test, which includes the culture of bacteria, takes more than 2 weeks to complete. Previous studies have reported that a nucleotide mutation in 23S rRNA domain V was observed in more than 95% of macrolide-resistant MAC strains (16, 17). Thus, rapid tests identifying those mutations might help to provide accurate therapy for MAC lung disease. Moreover, most clarithromycin-resistant strains have a point mutation at position 2058 or 2059 (wild type, A2058/A2059; Escherichia coli numbering), including the TA, GA, AG, CA, AC, and AT genotype mutants (12, 18, 19). We previously developed the amplification refractory mutation system (ARMS)-PCR method for determining the 23S rRNA mutations at positions 2058 to 2059 in MAC by using agarose gel electrophoresis (19). Since few medical institutions use agarose gel electrophoresis, it is hoped that a more user-friendly assay can be developed.

Recently, we developed some screening assays for the identification of severe acute respiratory syndrome coronavirus 2 (SARS-CoV-2) variants using conventional high-resolution melting (HRM) analysis (20–23). HRM analysis is a post-PCR genotyping technique based on the melting behavior of amplicons using a real-time PCR instrument. Real-time PCR is a general method for diagnosing various diseases and is used by many medical institutions. By modifying HRM analysis, in this study, we have developed a novel melting curve-based assay with nonfluorescent labeled probes to detect the 23S rRNA mutations at positions 2058 to 2059 in MAC. This assay can discriminate between clarithromycin-susceptible strains (AA genotype) and clarithromycin-resistant strains (TA, GA, AG, CA, AC, and AT genotypes) using real-time PCR.

## RESULTS

### Conventional HRM analysis without a specific probe.

**FIG 1:**
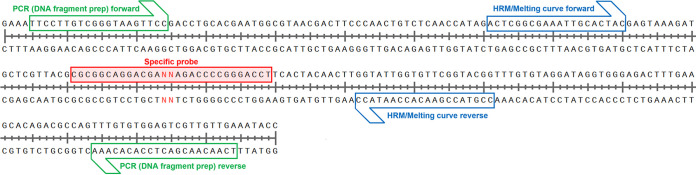
Partial sequence motif of 23S rRNA domain V from reference strains (Mycobacterium avium 104 and M. intracellulare ATCC 13950). The specific probes used in this study possess a genotype-specific sequence at NN (AA, TA, GA, AG, CA, AC, and AT).

Comparison of the genomic sequence data for M. avium strain 104 (GenBank accession no. NC_008595.1) and M. intracellulare strain ATCC 13950 (GenBank accession no. CP003322) revealed that M. avium and M. intracellulare possess the same sequences in the target region, including positions 2058 to 2059 of the 23S rRNA gene (Fig. 1). Hence, in this study, we used common primers and probes for the detection of clarithromycin-resistant M. avium and M. intracellulare strains (see Table S1 in the supplemental material). Prior to melting curve analysis with the specific probe, we performed a conventional HRM analysis using DNA fragments of seven genotypes (AA, TA, GA, AG, CA, AC, and AT). The DNA fragment of the AA genotype was distinguished from DNA fragments of GA, AG, CA, and AC genotypes by HRM analysis at positions 2058 to 2059 of the 23S rRNA gene (Fig. S1). However, there were slight differences between the DNA fragments of AA, TA, and AT genotypes. These results suggest that a conventional HRM assay alone can hardly discriminate between the AA genotype and other genotypes at positions 2058 to 2059 of the 23S rRNA gene.

### Melting curve analysis of DNA fragments using the AA-specific probe.

**FIG 2:**
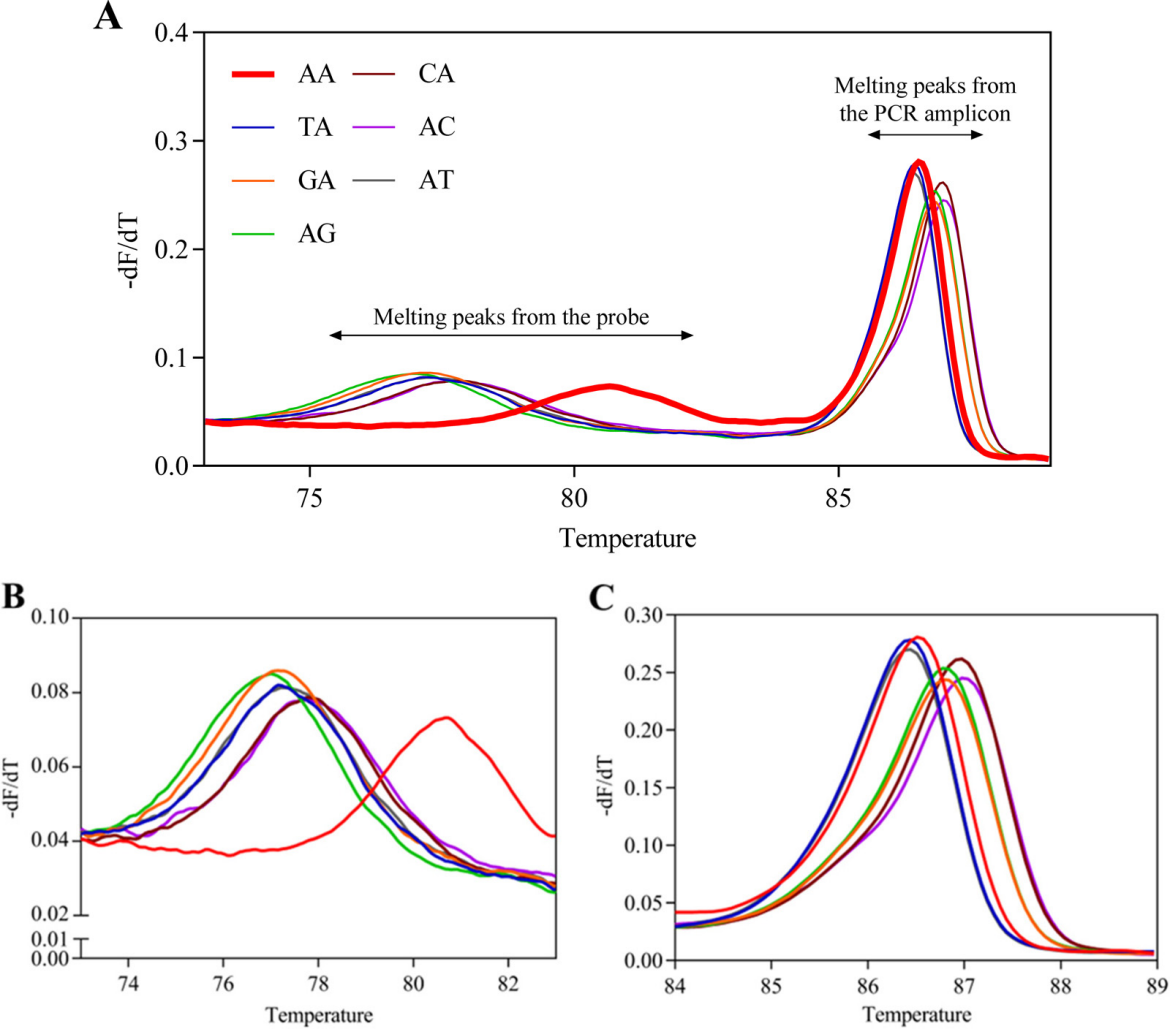
Melting peak plots of the DNA fragments of seven genotypes with the AA genotype-specific probe. Normalized melting peak plots (A) were acquired using DNA fragments of the AA (red line), TA (blue line), GA (orange line), AG (green line), CA (brown line), AC (purple line), and AT (gray line) genotypes. Each DNA fragment has two melting peaks derived from the probe (B) and the PCR amplicon (C).

At positions 2058 to 2059 of the 23S rRNA gene, the AA genotype MAC is the sole strain susceptible to clarithromycin, and six other genotype strains are resistant to clarithromycin (19). Therefore, we developed a screening assay to detect clarithromycin-resistant MAC using melting curve analysis with the AA-specific probe. Fig. 2 shows the melting peak plots of DNA fragments of seven genotypes by melting curve analysis using the AA-specific probe. The melting peak plots derived from PCR amplicons were higher than 84°C (Fig. 2C), similar to those in a conventional HRM assay. Since the 3′ end of the AA-specific probe was phosphorylated, asymmetric PCR amplified the short double-stranded DNA, which had a low melting peak below 82°C (Fig. 2B). The melting temperature (*T_m_*) value below 82°C of each melting curve plot was automatically calculated using Gene Scanning software. The *T_m_* value of the DNA fragment of the AA genotype (80.33 ± 0.10°C) was 2.5°C higher than that of the DNA fragments of all other genotypes at about 77°C (Table 1). These results suggest that the AA-specific probe’s melting curve assay can detect the 23S rRNA mutants (TA, GA, AG, CA, AC, and AT) according to the *T_m_* value of melting peaks at about 77°C.

**TABLE 1:** Melting temperature values of the DNA fragments of seven genotypes with specific probes

DNA fragment	*T_m_* for specific probe^*a*^						
	AA	TA	GA	AG	CA	AC	AT
AA	**80.33 ± 0.10**	77.33 ± 0.31	77.83 ± 0.11	77.58 ± 0.24	77.49 ± 0.03	77.22 ± 0.12	77.24 ± 0.07
TA	77.06 ± 0.13	**79.76 ± 0.23**	78.25 ± 0.09	75.78 ± 0.23	77.00 ± 0.05	75.69 ± 0.09	76.29 ± 0.06
GA	76.84 ± 0.14	77.02 ± 0.33	**81.12 ± 0.10**	76.84 ± 0.28	77.34 ± 0.03	75.71 ± 0.13	75.56 ± 0.07
AG	76.61 ± 0.13	75.27 ± 0.28	76.99 ± 0.19	**81.06 ± 0.20**	75.60 ± 0.04	76.97 ± 0.12	76.73 ± 0.10
CA	77.52 ± 0.07	77.92 ± 0.22	78.00 ± 0.11	75.98 ± 0.23	**81.77 ± 0.06**	77.25 ± 0.11	75.92 ± 0.08
AC	77.46 ± 0.08	76.19 ± 0.28	76.58 ± 0.18	77.81 ± 0.21	77.88 ± 0.07	**81.72 ± 0.09**	78.02 ± 0.13
AT	77.29 ± 0.05	76.23 ± 0.03	76.22 ± 0.09	78.56 ± 0.07	75.61 ± 0.02	76.61 ± 0.08	**79.75 ± 0.11**

a The melting temperature (*T_m_*) values are expressed as mean ± standard deviation of the triplicate independent assay. The highest *T_m_* values among each probe assay are in boldface.

### Melting curve analysis of DNA fragments using six other genotype-specific probes.

The AA-specific probe assay can discriminate between AA genotype and mutants; however, the AA-specific probe alone cannot determine the genotypes that each DNA fragment belongs to at positions 2058 to 2059 of the 23S rRNA gene. We then analyzed the melting curve with six other genotype-specific probes (TA, GA, AG, CA, AC, and AT). The melting peak plot of the TA genotype's DNA fragment had the highest *T_m_* value (79.76 ± 0.10°C) in TA-specific probe assays (Table 1 and Fig. S2A). In GA-, AG-, CA-, AC-, and AT-specific probe assays, the highest *T_m_* melting peaks were also obtained from the DNA fragments of identical genotypes to each specific probe (Table 1 and Fig. S2B to F). These results suggest that melting curve analysis using each genotype-specific probe can contribute to the identification of all genotypes at positions 2058 to 2059 of the 23S rRNA gene.

### Melting curve analysis of genomic DNA from MAC isolates.

Finally, we confirmed the applicability of this probe-based assay to genomic DNA from six genotype strains of M. avium and four genotype strains of M. intracellulare. As shown in Table 2, the clarithromycin susceptibility tests of 10 MAC strains used in this study showed that M. avium strain TH135 and M. intracellulare strain ATCC 13950 were clarithromycin-susceptible strains (MICs of ≤8 μg/mL), and five M. avium isolates and three M. intracellulare isolates were clarithromycin-resistant strains (MICs of ≥32 μg/mL). We then subjected these MAC strains to sequence analysis for positions 2058 to 2059 of the 23S rRNA gene. Two susceptible strains were wild type (AA genotype), while all eight resistant strains were mutants (TA, GA, AG, CA, or AC genotype).

**TABLE 2:** MICs of clarithromycin, sequence analysis for positions 2058 and 2059 of the 23S rRNA gene, and melting temperature values of DNA fragments from M. avium and M. intracellulare strains with specific probes

Strain	MIC (μg/mL)	Genotype	*T_m_* for specific probe^*a*^						
			AA	TA	GA	AG	CA	AC	AT
M. avium									
TH135	0.5	AA	**80.31**	77.03	77.66	77.51	77.55	76.99	77.45
TH26	>32	TA	76.91	**79.55**	78.21	75.72	77.05	75.47	76.35
TH136	>32	GA	76.78	76.74	**81.06**	76.96	77.33	75.52	75.65
CH-308	>32	AG	76.41	74.98	76.63	**81.06**	75.42	76.70	76.58
TH47	>32	CA	77.40	77.72	77.82	76.1	**81.78**	77.01	76.01
TH6	>32	AC	77.34	75.91	76.41	77.75	77.91	**81.63**	78.14
M. intracellulare									
ATCC 13950	0.03	AA	**80.21**	77.61	77.91	77.82	77.58	77.39	77.13
INT6	>32	TA	76.82	**80.04**	78.34	76.03	76.98	75.89	76.17
INT3	>32	GA	76.68	77.29	**81.42**	77.13	77.33	75.92	75.51
INT35	>32	CA	77.39	78.36	78.12	76.48	**82.02**	77.58	76.08

a The highest melting temperature (*T_m_*) values among each probe assay are in boldface.

M. avium and M. intracellulare possess the same sequence motif in part of 23S rRNA domain V, including positions 2058 to 2059, which were examined in this analysis (Fig. 1). Therefore, we investigated the applicability of the present melting curve-based assay for detecting mutations at positions 2058 to 2059 in both M. avium and M. intracellulare. Table 2 shows the *T_m_* values of the melting peaks from 10 genomic DNA by seven genotype-specific probe-based assays. In the AA-specific probe assay, the melting peaks of two genomic DNA from AA genotype isolates (M. avium strain TH135 and M. intracellulare strain ATCC 13950) agreed well with those of the DNA fragment of the AA genotype, and its *T_m_* value was about 80°C (Table 2). The *T_m_* values of the other genotype genomic DNA were about 77°C in the AA-specific probe assay. Through additional assays with other specific probes, all genomic DNAs can be correctly identified as individual genotypes according to the highest *T_m_* values among the same probe assays.

## DISCUSSION

This study developed a novel real-time PCR-based assay that detected the point mutations at positions 2058 to 2059 of 23S rRNA, thereby enabling identification of clarithromycin-resistant MAC. Sequence analysis, including the Sanger sequence and the whole-genome sequence, contributes to determining 23S rRNA mutations. However, sequence analysis is not high throughput and does not yield quick results. Previously, we developed a rapid assay based on ARMS-PCR for detecting 23S rRNA mutations at positions 2058 to 2059 in MAC (19). Although the ARMS-PCR method detected the 23S rRNA mutations with high accuracy, the method required the use of agarose gel electrophoresis. For medical institutions, a simpler and more user-friendly method is needed for determining 23S rRNA mutations. Real-time PCR is widely used as a high-throughput screening method for identifying point mutations in various areas of research because it has high sensitivity and is easy to use. In clinical practice, the diagnosis of MAC infection is based on real-time PCR, such as the Cobas TaqMan MAI (Roche Diagnostics). Hirama et al. (24) reported that a real-time PCR-based assay detected 23S rRNA mutations at positions 2058 to 2059 in MAC by using a dually labeled probe with a bridged nucleic acid (BNA). Using an AA genotype dually labeled BNA probe, the assay discriminated between the AA genotype strain and other strains. However, they did not determine the nucleotide sequence at positions 2058 to 2059 in mutant strains. In contrast, the present melting curve-based assay contributed to not only the detection of the MAC mutations but also the determination of all genotypes at positions 2058 to 2059 of the 23S rRNA gene. Additionally, the cost of running the present assay is lower than those of the BNA-based assay because we use a cost-effective and readily available probe, namely, a nonfluorescent labeled probe with 3′ phosphorylation. Therefore, our assay would be widely applicable in various medical institutions equipped with real-time PCR.

It is hoped that DNA from clinical samples in MAC patients could be used directly to detect clarithromycin resistance. According to the manufacturer's instructions, the TaqMan probe assay kit for diagnosing MAC infection, the Cobas TaqMan MAI (Roche Diagnostics), can detect 10^1^ copies of DNA/reaction. We investigated the present assay’s dynamic range using DNA fragments of seven genotypes (Table S2), which indicated that this assay could detect more than 10^2^ or 10^3^ copies/reaction. Thus, our melting curve-based assay may not detect clinical specimens with low copy numbers (less than 10^2^ copies/reaction) MAC. To identify the SARS-CoV-2 variants, we have successfully developed HRM-based assays with enough accuracy to detect low-copy-number specimens using the nested PCR method (20–23). Nested PCR consists of first (using an outer primer set) and second (using an inner primer set) rounds of amplification to improve an assay's detection limit and specificity. The nested PCR-based HRM assay could detect SARS-CoV-2 mutations of samples with 100- to 1,000-fold-lower copy numbers compared to the single HRM assay. Our preliminary study demonstrated that the nested PCR also improved the detection limit of the present melting curve-based assay, thereby enabling accurate detection of the 23S rRNA mutations in 10^1^ copies of DNA/reaction. These data suggest that our assay with nested PCR has enough sensitivity to detect the 23S rRNA mutations in clinical specimens diagnosed as MAC infection by the Cobas TaqMan MAI, although the clinical applicability needs to be evaluated by using clinical specimens, such as sputum from MAC patients, in actual clinical settings.

Given that the M. avium and M. intracellulare isolated from MAC patients are often clarithromycin-susceptible strains, the AA-specific probe assay can be applied as a general-use tool for detecting MAC mutation strains. When an additional assay is needed for genotyping mutant strains, other genotype-specific probes can contribute to the determination of genotypes. Moreover, melting curve plots derived from PCR amplicons (higher than 84°C) can help to identify genotypes (Fig. 2C). Based on plots of PCR amplicons, we can classify the mutants into three genotype groups: (i) TA or AT, (ii) GA or AG, and (iii) CA or AC.

The present melting curve-based assay focused on point mutations at positions 2058 to 2059 of the 23S rRNA gene in MAC strains. Although a point mutation at position 2058 or 2059 has been observed in many clarithromycin-resistant MAC strains, other point mutations in 23S rRNA domain V may also be involved in clarithromycin resistance (18, 24). Indeed, our previous studies demonstrated that one clarithromycin-resistant MAC strain had no mutation at positions 2058 to 2059 (19). These facts suggest that the detection of a mutation at positions 2058 to 2059 alone is not sufficient for identification of every clarithromycin-resistant MAC strain. A combination of multiple tests, including a culture-based clarithromycin susceptibility test and the present assay, may be necessary for identification of clarithromycin-resistant MAC strains.

Although this melting curve based-assay has some advantages over other assays, it also has limitations for the detection of clarithromycin-resistant MAC. First, this study demonstrated the assay using a limited number of MAC isolates. The AT genotype strain of M. avium and the AG, AC, and AT genotype strains of M. intracellulare were not investigated in this assay. More diverse strains must be analyzed to verify the usability of our assay for the detection of clarithromycin-resistant MAC. In addition, the present assay alone cannot identify all clarithromycin-resistant strains because there are some clarithromycin-resistant strains that have no mutations at positions 2058 to 2059 of the 23S rRNA gene. Second, this study did not directly analyze DNA from the clinical samples of MAC patients. Further studies are needed to confirm the utility of the present melting curve-based assay using large clinical samples to calculate the rate of false positives and false negatives. Third, we investigated all assays with a single real-time PCR instrument, the LightCycler 96. Our assay should be studied using other real-time instruments at several medical institutions.

In conclusion, we developed a novel melting curve-based assay for the detection of the 23S rRNA mutations at positions 2058 to 2059 in MAC, a key factor for clarithromycin-resistant M. avium and M. intracellulare, using real-time PCR. Furthermore, since nonfluorescent labeled probes are used, this assay has a lower running cost and is more readily available than other methods. This assay was not verified using clinical samples; therefore, further studies using diverse samples are needed to validate this melting curve-based assay in various medical institutions that have real-time PCR equipment.

## MATERIALS AND METHODS

### Bacterial strains.

M. avium strain 104 and M. intracellulare strain ATCC 13950 were used as reference strains (25). The clinical isolates used in this study were provided by Higashinagoya National Hospital of the National Hospital Organization in Aichi Prefecture, Japan. These clinical isolates consisted of six M. avium strains and three M. intracellulare strains isolated from patients diagnosed with MAC lung disease (corresponding to the diagnostic criteria of the American Thoracic Society and the Infectious Diseases Society of America [14]). All clinical isolates were identified as M. avium or M. intracellulare using the Cobas TaqMan MAI test (Roche Diagnostics, Basel, Switzerland) (26).

### Drug susceptibility testing.

As reported previously (27), BrothMIC NTM (Kyokuto Pharmaceutical Industrial Co., Ltd., Tokyo, Japan) was used to confirm the susceptibility of MAC strains to clarithromycin according to the manufacturer’s instructions (28). Based on the criteria described in the BrothMIC NTM manual, strains with a MIC for clarithromycin of ≤8 μg/mL were considered susceptible to clarithromycin and those with a MIC of ≥32 μg/mL were considered resistant to clarithromycin.

### Sequence analysis of DNA corresponding to domain V of the 23S rRNA gene.

The organism was grown in Middlebrook 7H9 liquid medium supplemented with 10% oleic acid-albumin-dextrose-catalase (OADC) enrichment (Difco, Sparks, MD) at 37°C. Genomic DNA from MAC isolates was extracted using InstaGene Matrix (Bio-Rad Laboratories, Hercules, CA) according to the manufacturer’s instructions. The resulting DNA (2 × 10^3^ to 7 × 10^3^ copies/reaction) was used for sequence and melting curve analyses. PCR was performed to amplify the region corresponding to domain V of the 23S rRNA gene using the primer pairs for the preparation of DNA fragments listed in Table S1 in the supplemental material. The PCR products were purified using spin columns (MinElute PCR purification kit; Qiagen GmbH, Hilden, Germany) and confirmed by sequence analysis (Eurofins Genomics KK, Tokyo, Japan). The resulting nucleotide sequences were compared with the genomic sequence data for M. avium strain 104 (GenBank accession no. NC_008595.1) and M. intracellulare strain ATCC 13950 (GenBank accession no. CP003322).

### Preparation of DNA fragments as the standard control.

According to the reference sequences (M. avium strain 104 and M. intracellulare strain ATCC 13950), seven synthetic DNA fragments (AA, TA, GA, AG, CA, AC, and AT genotypes; 240 bp in length) were obtained from Eurofins Genomics KK (Tokyo, Japan) and used for PCR amplification. DNA fragments were amplified by high-fidelity PCR (KOD Fx neo; Toyobo, Osaka, Japan) according to the manufacturer’s instructions (Fig. 1). The primer pairs used for PCR amplification are listed in Table S1. Each DNA fragment was observed as a single, correctly sized band (230 bp). The PCR products were purified using spin columns (MinElute PCR purification kit; Qiagen) and confirmed by sequence analysis (Eurofins Genomics KK). Purified DNA fragment products were used as the standard control for the HRM and melting curve analyses.

### HRM analysis.

HRM was performed using a high-fidelity PCR enzyme (TaKaRa *Ex Taq* HS; TaKaRa Bio, Shiga, Japan) and HRM dye (LightCycler 480 ResoLight dye; Roche Diagnostics). Each reaction mixture (10 μL) contained 1 μL of the purified DNA fragments (1 × 10^4^ copies/reaction), 400 nmol/L of each primer (Table S1), 3 mmol/L of MgCl_2_, 200 μmol/L of deoxynucleoside triphosphates (dNTPs), 0.025 U/μL of *Ex Taq* HS enzyme, 1× ResoLight dye, and 1× PCR buffer. All reactions were duplicated using a LightCycler 96 real-time PCR system (Roche Diagnostics). Symmetric PCR amplification was performed with an initial denaturation at 95°C for 5 min, followed by 45 cycles of denaturation at 95°C for 10 s, annealing at 60°C for 10 s, and extension at 72°C for 20 s. After amplification, HRM was performed with denaturation at 95°C for 60 s, cooling at 40°C for 60 s, preheating at 65°C for 1 s, and melting curve generation from 65°C to 95°C in 1°C/s increments with 25 acquisitions. Under default settings, HRM curves were analyzed using Gene Scanning software, version 1.1.0.1320 (Roche Diagnostics). Normalized melting peaks (−dF/dT) were acquired by setting premelt and postmelt fluorescence levels to 100% and 0%, respectively. After HRM analysis, each DNA fragment was observed as a single, correctly sized band (101 bp) by electrophoresis.

### Melting curve analysis with nonfluorescent labeled probes.

Seven genotype-specific probes with 3′ phosphorylation were obtained from Eurofins Genomics KK and are listed in Table S1. Melting curve analysis was performed under the modified HRM condition for asymmetric PCR. Each reaction mixture (10 μL) contained 1 μL of the purified DNA fragment (1 × 10^4^ copies/reaction), 360 nmol/L of each genotype-specific probe, 40 nmol/L of forward primer, 400 nmol/L of reverse primer, 3 mmol/L of MgCl_2_, 200 μmol/L of dNTPs, 0.025 U/μL of *Ex Taq* HS enzyme, 1× ResoLight dye, and 1× PCR buffer. All reactions were duplicated using a LightCycler 96 real-time PCR system. Asymmetric PCR amplification was performed with an initial denaturation at 95°C for 5 min, followed by 45 cycles of denaturation at 95°C for 10 s, annealing at 60°C for 10 s, and extension at 72°C for 20 s. After amplification, melting curve analysis was performed with denaturation at 95°C for 60 s, cooling at 40°C for 60 s, preheating at 65°C for 1 s, and melting curve generation from 65°C to 95°C in 1°C/s increments with 25 acquisitions. Normalized melting peaks (−dF/dT) and *T_m_* values were acquired using Gene Scanning software version 1.1.0.1320.

### Ethics statement.

This project was approved by the Ethics Review Committee for Human Research of Higashinagoya National Hospital of the National Hospital Organization, and written informed consent was obtained from all patients.
