# Supplementary material for: A Rapid Screening Assay for Clarithromycin-Resistant Mycobacterium avium Complex Using Melting Curve Analysis with Nonfluorescent Labeled Probes
Source: Microbiol Spectr. 2023 Jan 9;11(1):e04326-22. doi: 10.1128/spectrum.04326-22 (PMC9927575; doi:10.1128/spectrum.04326-22)
Supplement: Supplemental file 1 — Supplemental material. Download spectrum.04326-22-s0001.pdf, PDF file, 1.3 MB [file spectrum.04326-22-s0001.pdf]

Supplemental Material

Supplemental figures

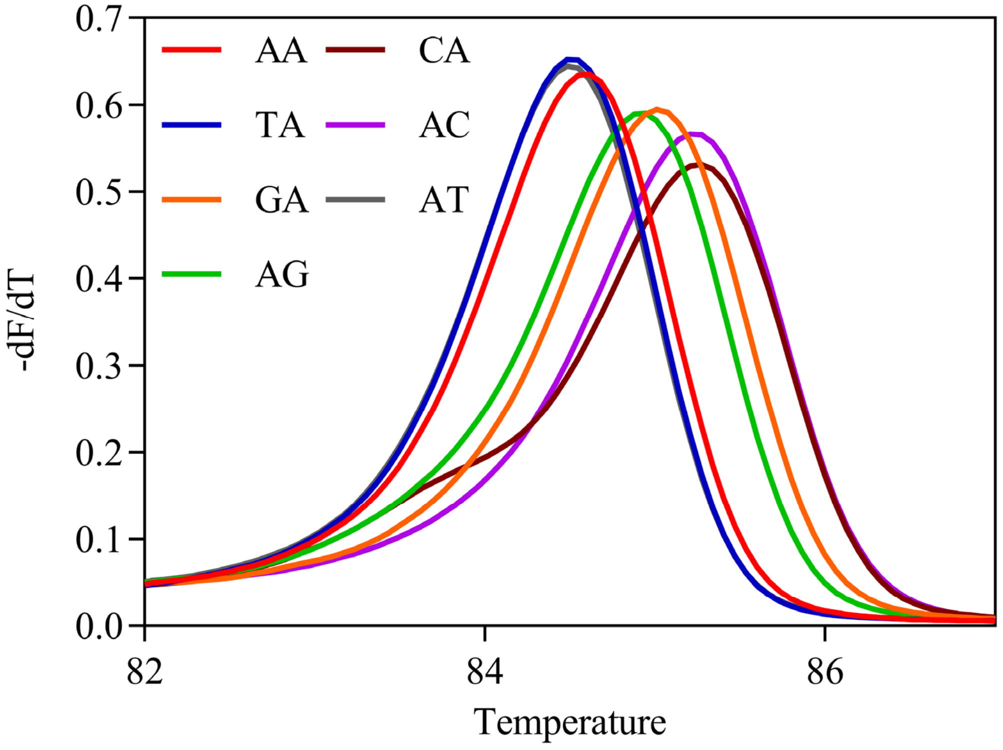

Figure S1 Melting peak plots of the DNA fragments of seven genotypes by a conventional high-resolution melting analysis. Normalized melting peak plots were acquired using DNA fragments of the AA (red line), TA (blue line), GA (orange line), AG (green line), CA (brown line), AC (purple line), and AT (gray line) genotypes.

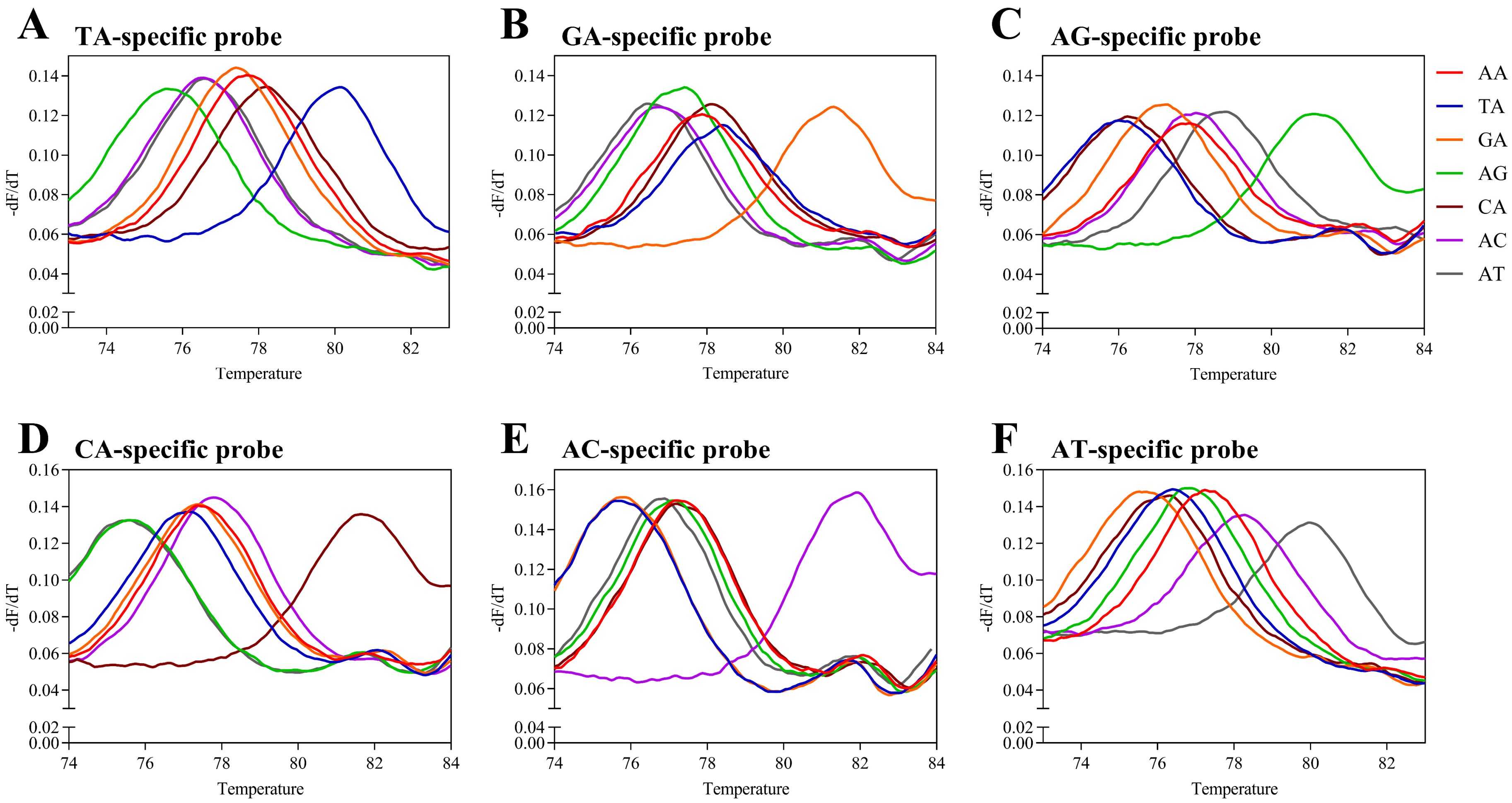

Figure S2 Melting peak plots of the DNA fragments of seven genotypes derived from the probe using the TA-specific probe (A), GA-specific probe (B), AG-specific probe (C), CA-specific probe (D), AC-specific probe (E), and AT-specific probe (F).

Supplemental Tables

Table S1 Sequences for primers and probes in PCR for the preparation of DNA fragments, high-resolution melting (HRM), and melting curve analyses.

Primer/probe	Sequence
PCR for the preparation of DNA fragments	
Fwd primer	5'-TTCCTTGTCGGGTAAGTTCC-3'
Rev primer	5'-TCAACAACGACTCCACACAAA-3'
HRM and melting curve analyses	
Fwd primer	5'-ACTCGGCGAAATTGCACTAC-3'
Rev primer	5'-CCGTACCGAACACCAATACC-3'
Probe[a]	5'-CGCGGCAGGACGANN[a]AGACCCCGGGACCT-3'[Pho]

[a]Each probe possesses a genotype-specific sequence at NN (AA, TA, GA, AG, CA, AC, and AT).
Fwd: forward; Rev: reverse; Pho: phosphorylation.

Table S2 Melting temperature values derived from the probe using 10-fold serial dilutions of the DNA fragments of seven genotypes.

Copies/ reaction	Probe						
	AA	TA	GA	AG	CA	AC	AT
10^7	79.97	79.56	81.28	81.56	81.81	81.75	79.20
10^6	79.96	79.5	81.23	81.51	81.77	81.97	79.42
10^5	80.05	79.49	81.25	81.57	81.83	81.99	79.53
10^4	80.13	79.51	81.26	81.58	81.83	82.06	79.75
10^3	80.08	79.44	81.26	81.56	81.86	82.06	79.81
10^2	79.81	79.45	77.16	78.14	81.88	82.02	79.21
10^1	77.37	76.45	77.27	78.01	77.81	77.26	77.72
10^0	77.19	76.67	77.28	78.11	77.90	77.25	77.58
